# Common childhood vaccines do not elicit a cross-reactive antibody response against SARS-CoV-2

**DOI:** 10.1371/journal.pone.0241471

**Published:** 2020-10-28

**Authors:** Ahmed Kandeil, Mokhtar R. Gomaa, Ahmed El Taweel, Ahmed Mostafa, Mahmoud Shehata, Ahmed E. Kayed, Omnia Kutkat, Yassmin Moatasim, Sara H. Mahmoud, Mina Nabil Kamel, Noura M. Abo Shama, Mohamed El Sayes, Rabeh El-Shesheny, Mahmoud A. Yassien, Richard J. Webby, Ghazi Kayali, Mohamed A. Ali

**Affiliations:** 1 Center of Scientific Excellence for Influenza Viruses, National Research Centre, Giza, Egypt; 2 Biological Sector, Egyptian Drug Authority, Cairo, Egypt; 3 Department of Infectious Diseases, St. Jude Children’s Research Hospital, Memphis, Tennessee, United States of America; 4 Department of Epidemiology, Human Genetics, and Environmental Sciences, University of Texas, Houston, Texas, United States of America; 5 Human Link, Hazmieh, Baabda, Lebanon; University of South Dakota, UNITED STATES

## Abstract

Anecdotal evidence showed a negative correlation between Bacille Calmette-Guérin (BCG) vaccination and incidence of COVID-19. Incidence of the disease in children is much lower than in adults. It is hypothesized that BCG and other childhood vaccinations may provide some protection against SARS-CoV-2 infection through trained or adaptive immune responses. Here, we tested whether BCG, Pneumococcal, Rotavirus, Diphtheria, Tetanus, Pertussis, Hepatitis B, Haemophilus influenzae, Hepatitis B, Meningococcal, Measles, Mumps, and Rubella vaccines provide cross-reactive neutralizing antibodies against SARS-CoV-2 in BALB/c mice. Results indicated that none of these vaccines provided antibodies capable of neutralizing SARS-CoV-2 up to seven weeks post vaccination. We conclude that if such vaccines have any role in COVID-19 immunity, this role is not antibody-mediated.

## Introduction

A novel coronavirus, Severe Acute Respiratory Syndrome Coronavirus 2 (SARS-CoV-2), has emerged during December 2019 in Wuhan, China. It is the causative agent for human coronavirus disease 2019 (COVID-19). Common symptoms caused by SARS-CoV-2 include fever, cough, and shortness of breath. Other symptoms may develop including production of sputum, muscle pain, diarrhea, sore throat, loss of smell, loss of taste and abdominal pain [1]. According to the World Health Organization’s situation report number 182 (July 20, 2020), more than 14 million cases were reported and more than 600,000 died while most of the detected cases have mild symptoms [2]. The highest incidence of SARS-CoV-2 infection occurs in older people and case fatality rate increases with age [3, 4]. The rates of infection and mortality were the lowest among patients aged 0–20 years. Also, the symptoms among younger infected patients are mild compared to older patients [5]. This phenomenon has been globally observed. Contrary to this evidence, researches indicate that young people do not have immune systems as efficient as adults. One assumption for the lower SARS-CoV-2 infectivity in children is the cross-reactive antibodies that are provoked in children as a response to one or more of their childhood vaccines. Another concept for children sparing by infection with SARS-CoV-2 might be the low immunity in childhood that does not inflate host immunity defense against the virus as in adults [6]. Similar to the current pandemic, children were less affected and had lower mortality during the SARS-CoV-1 and MERS-CoV outbreaks [7].

In 2015, about 85% of children worldwide were subjected to vaccination for tuberculosis, diphtheria, tetanus, polio, pertussis, and measles [8]. Since 2010, about 235 million children were immunized for meningitis A in an area that covers 26 countries in sub-Saharan Africa. According to the CDC, children from 1–2 months should receive Hepatitis B (HepB), Diphtheria, tetanus, and whooping cough (pertussis) (DTaP), Haemophilus influenzae type b (Hib), Polio (IPV), Pneumococcal (PCV), and Rotavirus (RV). At 4 months of age, children should receive DTaP, Hib, IPV, PCV, RV, and HepB. At 6 months of age, children should receive the previous vaccines except HepB. By following the recommended schedule of CDC, child during 1 to 2 years of age should be immunized against 14 vaccine-preventable diseases including Chickenpox (Varicella), DTaP, Hib, MMR, IPV, PCV, Hepatitis A (HepA) and HepB. Between 4 through 6 years of age, children should be vaccinated against DTaP, IPV, MMR, Chickenpox (varicella) and influenza. Bacille Calmette-Guérin (BCG) is a common vaccine for tuberculosis (TB) disease. This vaccine is not widely used in the developed countries, but it is often given to children in some of developing countries where TB is common. Most of routine vaccines are either inactivated or live attenuated vaccines. Live attenuated vaccines elicit strong cellular and humoral immune response contrary to inactivated vaccines that stimulate humoral immunity. Cross reactivity between vaccination and heterologous virus strains has been observed when serum antibodies against HIV emerged after measles vaccination [9].

Several hypotheses emerged on the potential of childhood immunization and BCG vaccination to offer protection against SARS-CoV-2 through trained immunity [10]. One hypothesis is that humoral immunity initiated by MMR vaccination may protect against COVID-19 [11]. Observational studies concluded that countries with BCG vaccination programs had significantly improved COVID-19 outcomes [12–16]. Here, we investigated whether common childhood vaccines and BCG played a role in antibody mediated immune response against COVID-19 by testing whether those vaccines produced cross-reactive neutralizing antibodies against SARS-CoV-2.

## Materials and methods

### Childhood vaccines

Seven of the most common childhood vaccines including Pneumococcal polysaccharide conjugate vaccine (Pfizer, New York, New York, USA), Rotavirus (GlaxoSmithKline, Brentford, UK), Pentavalent vaccine of Diphtheria, Tetanus, Pertussis, Hepatitis B, and Haemophilus influenzae type b Conjugate Vaccine Adsorbed (Serum Institute of India, Pune, India), Hepatitis B Vaccine (Serum Institute of India), Meningococcal Conjugate Vaccine (Sanofi Pasteur, Lyon, France), Measles, Mumps and Rubella (MMR) live attenuated vaccine (GlaxoSmithKline), and BCG vaccine (Green Signal Biopharma, Chennai, India) were kindly obtained from the Egyptian Drug Authority.

### SARS-CoV-2 inactivated vaccine

A hCoV-19/Egypt/NRC-03/2020 SARS-CoV-2 strain (GISAID accession number: EPI_ISL_430820) was isolated in VeroE6 cells. The virus-infected culture supernatant was clarified by centrifugation at 4,000 rpm for 15 min at 4 °C twice. Formalin inactivation was done at a concentration of 0.1% and kept for 2 days at 32 °C. Formalin-treated virus was tested for its infectivity in cells. Inactivated virus was concentrated using ultracentrifugation. The pellet was resuspended in PBS and total protein content was measured using nanodrop spectrophotometer. A volume of 3 ml of antigen was mixed with 3 ml of alum adjuvant. To assess the immunogenicity of the inactivated vaccine as a positive control, 6 BALB/c mice were intramuscularly injected at day 0 and 21 (15 μg/dose).

### Immunization of mice

**Fig 1:**
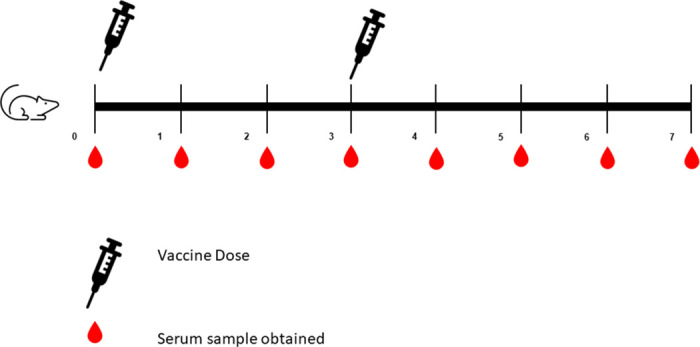
Experimental timeline of immunization of mice of tested childhood vaccines and inactivated SARS-CoV-2 vaccine. A syringe indicate a vaccination time point, a drop indicates a serum sample time point.

Female BALB/c mice (6-8-week-old) were obtained from Animal House at National Research Centre (NRC), Egypt. Mice were divided into nine groups (5 mice/group). Two groups of mice were orally and intradermally vaccinated with 200 μl of live rotavirus and BCG vaccines, respectively. The other remaining groups were intramuscularly injected with 200 μl of each type of tested vaccines. A negative control group was injected with sterile PBS. All vaccinated animals received booster immunization doses after 3 weeks of the first dose. Serum samples were weekly collected till the 7^th^ week of immunization (Fig 1).

### Microneutralization assay (MN)

The MN was conducted as previously described using Vero-E6 cell with minor modifications [17]. Briefly, serial two-fold dilutions of heat-inactivated serum samples starting with a dilution of 1:10 in infection medium were mixed with equal volumes of 100 tissue culture infectious dose 50 (TCID50/mL) of an hCoV-19/Egypt/NRC-03/2020 SARS-CoV-2 isolate. After an hour post incubation at 37 °C, 35 μl of the virus–serum mixture were added in duplicate to Vero-E6 cells in 96-well tissue culture plates. After 1 h of infection, the inoculums were removed and 150 μl of infection medium was added to each well. The plates were then incubated for three days at 37 °C in a humidified CO_2_ incubator. Cytopathic effect (CPE) was recorded for each plate. The highest serum dilution that prevented virus to develop CPE was recorded as the neutralizing antibody titer.

### Human sera

One hundred and forty serum samples previously collected during 2019 of our ongoing cohort study to determine household transmission of zoonotic influenza viruses were categorized by age (2–10 years old, 10–20 years old, 20–30 years old, 30–40 years old., 40–50 years old, 50–60 years old, >60 years old) (20 serum samples/category). Serum samples were processed for serological testing against SARS-CoV2 using MN assay.

### Ethics statement

The animal experiment was conducted at NRC based on national and international animal welfare guidelines. The Research Ethics Committee of the NRC approved the animal experiment in mice under approval number 20074.

## Results

**Fig 2:**
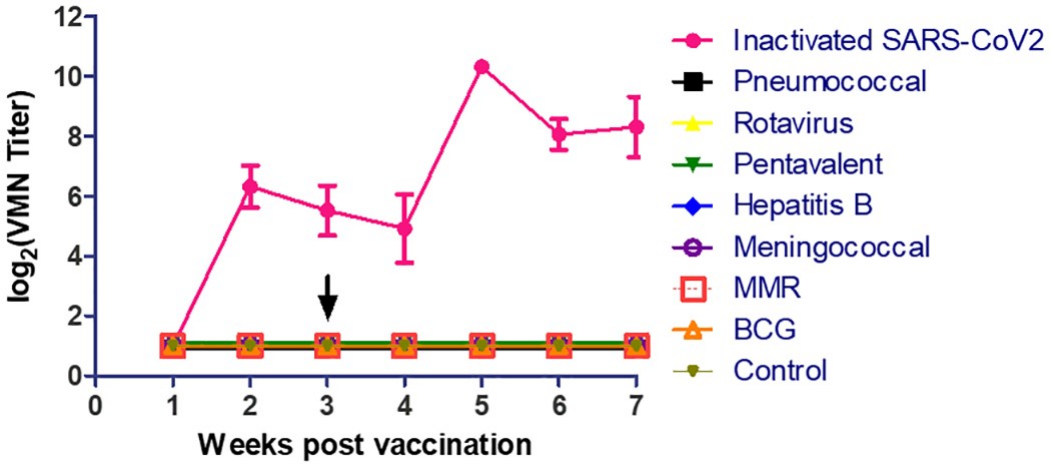
Weekly follow up of VMN titer against SARS-CoV-2 in immunized mice with most common childhood vaccines and inactivated SARS-CoV-2 vaccine.

The results are shown in Fig 2. None of the childhood vaccines tested in this study elicited an antibody response in vaccinated mice throughout the duration of the experiment. In contrast, the inactivated SARS-CoV-2 vaccine provided a detectable neutralizing antibody titer as of week 2 post vaccination reaching an average of 6 log2 (Wilcoxon’s Sum Rank Test p-value < 0.05). This titer began to decline at weeks 3 and 4 post vaccination but reached an average of 10 log2, 2 weeks after the mice received the booster dose. An average titer of around 8 log2 continued to be observed during weeks 6 and 7 post vaccination.

None of the tested human serum samples collected during 2019 of different ages showed cross-reactivity with SARS-CoV-2.

## Discussion

Here we aimed to study whether BCG and other common childhood vaccines can elicit cross-reactive antibodies against SARS-CoV-2. None of the tested vaccines elicited such a response while the positive control inactivated SARS-CoV-2 vaccine provided relatively high neutralizing antibody titers.

Several reports correlated lower incidence of COVID-19 to using BCG vaccine [12–16]. Previous research has shown that BCG may induce trained immunity providing a better immune response to heterologous pathogens through various mechanisms affecting cellular immunity [18–20]. The vaccine enhanced production of pro-inflammatory cytokines, such as IL-1β, tumor necrosis factor, and IL-6 [21]. However, the role of BCG against COVID-19 remains unclear and requires further experiments and clinical trials [22]. A cohort study found no difference in SARS-CoV-2 infection rates among BCG-vaccinated versus unvaccinated adults [23]. Our data showed that BCG vaccination does not provide cross-reactive antibodies but does not negate a potential role in cellular immunity.

The role of childhood vaccination in COVID-19 is even less clear than that of BCG. It is hypothesized that frequent childhood vaccinations and repeated infections might result in trained immunity, better immune fitness of adaptive immune cells, or cross-protection of antibodies in the children [6]. Our data show that Pneumococcal, Rotavirus, Diphtheria, Tetanus, Pertussis, Hepatitis B, Haemophilus influenzae, Hepatitis B, Meningococcal, and MMR vaccines do not provide cross-protective neutralizing antibodies against COVID-19.

Whether childhood vaccines have a role in protection against COVID-19 through other immune mechanisms remains unclear. Correlation among those vaccines and SARS-CoV-2 infection could be investigated further to identify potential cellular and cytokine responses.

In summary, BCG and common pediatric vaccines do not provide neutralizing antibodies against SARS-CoV-2 and whether they play a role through other immune response pathways requires further investigations.

## Supporting information

S1 Table Raw data of weekly follow up of VMN titer against SARS-CoV-2 in immunized mice with most common childhood vaccines and inactivated SARS-CoV-2 vaccine. (XLSX)
